# The EANM practice guidelines for bone scintigraphy

**DOI:** 10.1007/s00259-016-3415-4

**Published:** 2016-06-04

**Authors:** T. Van den Wyngaert, K. Strobel, W. U. Kampen, T. Kuwert, W. van der Bruggen, H. K. Mohan, G. Gnanasegaran, R. Delgado-Bolton, W. A. Weber, M. Beheshti, W. Langsteger, F. Giammarile, F. M. Mottaghy, F. Paycha, On behalf of the EANM Bone & Joint Committee and the Oncology Committee.

**Affiliations:** Department of Nuclear Medicine, Antwerp University Hospital, Wilrijkstraat 10, 2650 Edegem, Belgium; Faculty of Medicine and Health Sciences, University of Antwerp, Universiteitsplein 1, 2610 Wilrijk, Belgium; Department of Radiology and Nuclear Medicine, Lucerne Cantonal Hospital, Lucerne, Switzerland; Nuclear Medicine Spitalerhof, Spitalerstraße 8, 20095 Hamburg, Germany; Clinic of Nuclear Medicine, Friedrich-Alexander-University Erlangen-Nürnberg, Erlangen, Germany; Department of Radiology and Nuclear Medicine, Slingeland Hospital, Doetinchem, The Netherlands; Department of Nuclear Medicine, Guy’s and St Thomas’ NHS Foundation Trust, London, UK; Department of Nuclear Medicine, Royal Free London NHS Foundation Trust, London, UK; Department of Diagnostic Imaging (Radiology) and Nuclear Medicine, San Pedro Hospital and Centre for Biomedical Research of La Rioja (CIBIR), University of La Rioja, Logroño, La Rioja Spain; Department of Radiology, Memorial Sloan Kettering Center, New York, NY USA; PET-CT Center Linz, Department of Nuclear Medicine and Endocrinology, St Vincent’s Hospital, Seilerstaette 4, 4020 Linz, Austria; Department of Nuclear Medicine, Centre Hospitalier Universitaire de Lyon, Lyon, France; Department of Nuclear Medicine, University Hospital Aachen, RWTH Aachen University, Aachen, Germany; Department of Nuclear Medicine, Maastricht University Medical Center (MUMC+), Maastricht, The Netherlands; Department of Nuclear Medicine, Hôpital Lariboisière, Assistance Publique-Hôpitaux de Paris, 2 rue Ambroise Paré, 75010 Paris, France

**Keywords:** Bone scintigraphy, Bone scan, Bone SPECT/CT, Bone disease

## Abstract

**Purpose:**

The radionuclide bone scan is the cornerstone of skeletal nuclear medicine imaging. Bone scintigraphy is a highly sensitive diagnostic nuclear medicine imaging technique that uses a radiotracer to evaluate the distribution of active bone formation in the skeleton related to malignant and benign disease, as well as physiological processes.

**Methods:**

The European Association of Nuclear Medicine (EANM) has written and approved these guidelines to promote the use of nuclear medicine procedures of high quality.

**Conclusion:**

The present guidelines offer assistance to nuclear medicine practitioners in optimizing the diagnostic procedure and interpreting bone scintigraphy. These guidelines describe the protocols that are currently accepted and used routinely, but do not include all existing procedures. They should therefore not be taken as exclusive of other nuclear medicine modalities that can be used to obtain comparable results. It is important to remember that the resources and facilities available for patient care may vary.

## Preamble

The aim of this document is to provide general information about bone scintigraphy. This guidelines describe current routine clinical procedures but should not be interpreted as excluding alternative procedures also employed to obtain equivalent data. It is important to remember that the resources and facilities available for patient care may vary from one country to another and from one medical institution to another. This document has been prepared primarily for nuclear medicine physicians, physicists and technicians with the intention of offering assistance in optimizing procedural protocols and diagnostic information that can currently be obtained from bone scintigraphy. The guidelines were written by the EANM Bone & Joint and Oncology Committees and then reviewed and approved by all EANM committees and the Board, as well as by the National Societies represented within the EANM.

The EANM wrote and approved the guidelines to promote the use of high-quality nuclear medicine procedures. These guidelines are intended to assist practitioners in providing appropriate nuclear medicine care for patients. They are not inflexible rules or requirements for practice and are not intended, nor should they be used, to establish a legal standard of care. For these reasons and those set out below, the EANM cautions against the use of these guidelines in litigation in which the clinical decisions of a practitioner are called into question.

The ultimate judgment regarding the propriety of any specific procedure or course of action must be made by medical professionals taking into account the unique circumstances of each case. Thus, an approach that differs from the guidelines does not necessarily imply that the approach was below the standard of care. To the contrary, a conscientious practitioner may responsibly adopt a course of action different from that set out in the guidelines when, in the reasonable judgment of the practitioner, such course of action is indicated by the condition of the patient, the limitations of available resources, or advances in knowledge or technology subsequent to publication of the guidelines.

The practice of medicine involves not only the science, but also the art of dealing with the prevention, diagnosis, alleviation, and treatment of disease. The variety and complexity of human conditions make it impossible at times to identify the most appropriate diagnosis or to predict with certainty a particular response to treatment. Therefore, it should be recognized that adherence to these guidelines will not assure an accurate diagnosis or a successful outcome. All that should be expected is that the practitioner will follow a reasonable course of action based on current knowledge, available resources, and the needs of the patient to deliver effective and safe medical care. The sole purpose of these guidelines is to assist practitioners in achieving this objective.

## Introduction and goals

The radionuclide bone scan is the cornerstone of skeletal nuclear medicine imaging. Bone scintigraphy is a highly sensitive diagnostic nuclear medicine imaging technique that uses a radiotracer to evaluate the distribution of active bone formation in the skeleton related to malignant and benign diseases, as well as physiological processes. Phosphate analogues can be labelled with technetium-99m (^99m^Tc) and are used for bone imaging because of their high uptake in the skeleton and rapid clearance from soft tissues after intravenous injection. Tracer accumulation occurs in proportion to local blood flow and bone remodelling activity (dependent on osteoblast/osteoclast activity), and unbound tracer is rapidly cleared from surrounding soft tissues.

Most pathological bone conditions, whether of infectious, traumatic, neoplastic or other origin, are associated with an increase in vascularization and local bone remodelling. This accompanying bone reaction is reflected on bone scans as a focus of increased radioactive tracer uptake. Bone scintigraphy is a sensitive technique that can detect significant metabolic changes very early, often several weeks or even months before they become apparent on conventional radiological images. In addition, the technique provides an overview of the entire skeleton at a relatively modest radiation exposure.

While MRI has been shown to be more sensitive than planar bone scans in detecting skeletal metastases in vertebral bodies, bone SPECT has been found to have comparable diagnostic sensitivity for vertebral body metastases and a higher diagnostic sensitivity for metastases located in the pedicles [1]. Furthermore, the majority of studies that have compared bone scanning with MRI did not use a reliable gold standard and typically included various solid tumours and even lymphomas which usually produce osteolytic lesions with poor osteoblastic bone reaction so that they are not detectable by bone scintigraphy. Hence, there is no reliable evidence that bone scintigraphy is generally less sensitive than whole-body MRI in all solid cancers. Multimodality SPECT/CT offers the unique opportunity to correlate scintigraphic findings with anatomical images and introduces novel algorithms to further enhance SPECT image quality based on the CT data (e.g. correction for attenuation and scatter). This results in improved correlation of areas with physiological variants or abnormal tracer accumulation with anatomical landmarks [2]. However, this has increased the complexity of this technique, increasing the need for standardization and practice guidelines in order to maximize the diagnostic yield of the examination.

The corresponding guidelines from the Society of Nuclear Medicine and Molecular Imaging (SNMMI), the Dutch Society of Nuclear Medicine (NVNG), and the French Society of Nuclear Medicine and Molecular Imaging (SFMN), as well as the existing EANM bone scintigraphy procedures guideline for tumour imaging were consulted while preparing this consensus document [3–6]. For specific applications of bone scintigraphy in selected indications or populations, the reader is also referred to the EANM guidelines for paediatric bone scanning, and the EANM and SNMMI practice guidelines for sodium ^18^F-fluoride PET/CT bone scans [7–9]. Other excellent reference works are also recommended [10].

The goal of these guidelines is to provide an educational tool designed to assist the nuclear medicine practitioner in appropriately recommending, performing, interpreting, and reporting the results of bone scintigraphy.

## Definitions

1. *Planar whole-body images* in the anterior and posterior projections of the axial and appendicular skeleton. If necessary, additional localized or spot views can be obtained.
2. *Focal planar images* limited to a specific portion of the skeleton.
3. *Single photon emission tomography (SPECT, also known as SPET)* allows the visualization of the three-dimensional distribution of the radiopharmaceutical in the skeleton.
4. *SPECT/CT images* consist of a SPECT acquisition combined with CT using an integrated CT scanner.
5. *Multiphase bone scan* produces planar images of the vascular inflow and the soft tissue phase, and delayed phase images of the radiopharmaceutical over a given area of the skeleton. The vascular inflow images are acquired during intravenous injection. The study of the soft tissue distribution of the radiopharmaceutical in the region of interest is performed within the first 5 – 10 min after injection. Finally, delayed whole-body, focal views, and/or tomographic images are usually acquired between 2 and 4 h after injection of the radiopharmaceutical. In some patients, it may be useful to acquire late-phase images up to 24 h after tracer administration [11].
6. *Quantification* is the process of calculating the osseous radioactivity concentration expressed as standardized uptake values (SUV).

## Common clinical indications for bone scan

The indications for bone scintigraphy are numerous and can generally be classified into three distinct clinical scenarios: (1) when a specific bone disease is present or suspected, (2) to explore unexplained symptoms, and (3) for the metabolic assessment prior to the start of therapy. While the diagnostic sensitivity of bone scintigraphy is very high, the low specificity often requires further investigation with other imaging modalities (e.g. plain radiography, CT or MRI) or nuclear medicine studies (e.g. FDG PET/CT). For this reason, anatomical imaging and bone scintigraphy should be considered as complementary methods, each of which cannot be replaced by the other.

Conversely, bone scintigraphy is not indicated in a number of specific conditions due to limitations of the technique in a specific disease context or lack of clinical impact of imaging results. However, ultimately it must be the nuclear medicine physician who evaluates and decides on the indication in each particular case and on the specific protocol that should be applied. Both indications and nonrecommended indications are outlined in the following sections.

### Bone disease present or suspected

1. Oncology
  Solid tumours with high affinity for bone, including prostate, breast, lung and renal cancer [12–17]
  Malignant haematological conditions limited to bone, including Hodgkin’s disease and non-Hodgkin’s lymphoma [18]
  Bone tumours and bone dysplasia, including osteosarcoma, osteoid osteoma, osteoblastoma, fibrous dysplasia, giant cell tumour and osteopoikilosis [19]
  Soft tissue sarcomas, including rhabdomyosarcoma
  Paraneoplastic syndromes, including hypertrophic pulmonary osteoarthropathy, algodystrophy, polymyalgia rheumatica, poly(dermato)myositis and osteomalacia
  Assessment of bone remodelling prior to radionuclide therapy (^223^Ra-Cl_2_, ^89^Sr-Cl_2_, ^153^Sm-EDTMP, ^186^Re-HEDP)
  Whole-body FDG PET and PET/CT are other imaging modalities that enable detection of the primary tumour and metastases by visualization of the increased glucose consumption of malignant tissue. The majority of studies comparing FDG PET with ^18^F-fluoride PET or SPECT with ^99m^Tc-labelled bisphosphonates have shown a higher diagnostic sensitivity of FDG for osteolytic metastases and a higher diagnostic sensitivity of bone-affine radiotracers for detecting osteoblastic metastases [20–24].
  Primary tumours of bone are relatively rare in adults, whereas bone metastases of other cancer entities (e.g. breast, prostate, lung, renal cancer, etc.) are very frequent. In prostate cancer, the assessment of bone scans is increasingly standardized by calculation of the bone scan index and reporting of progression according to PCWG-2 criteria [25, 26].
2. Rheumatology
  Chronic inflammatory arthritis, including rheumatoid arthritis, spondyloarthropathies and related disorders (ankylosing spondylitis, psoriatic arthritis, Reiter’s arthritis, SAPHO syndrome [synovitis, acne, pustulosis, hyperostosis, osteitis], chronic recurrent multifocal osteomyelitis) and sacroiliitis [27–29]
  Osteoarthritis of the lumbar facet joints, hip, femorotibial and femoropatellar osteoarthritis, rhizarthrosis and tarsal osteoarthritis [30, 31]
  Enthesopathies, including plantar fasciitis, Achilles tendinitis and bursitis
  (Avascular) osteonecrosis, which is most frequently located at the femoral head, femoral condyle and tibial plateau
  Osteonecrosis of the jaw (ONJ) [32]
  Complex regional pain syndrome type I of the hand, hip, knee and foot
  Tietze’s syndrome (costochondritis)
  Polymyositis
  Paget’s disease
  Langerhans cell histiocytosis (LCH): single system LCH and multisystem LCH with bone involvement
  Non-Langerhans cell diseases, such as Erdheim–Chester disease, Schnitzler syndrome, and Rosaï Dorfman disease
  Other rare osteoarticular diseases, such as sarcoidosis with bone involvement, mastocytosis, Behçet’s disease, and familial Mediterranean fever
3. Bone and joint infections [33]
  Osteomyelitis (acute, subacute or chronic, of bacterial, mycobacterial or fungal origin)
  Septic arthritis
  Spondylodiscitis or spondylitis
  Septic loosening or mechanical complication of internal fixation (long bones or spine) or arthroplasty (hip, knee, ankle or shoulder)
  Malignant (necrotizing) external otitis
  The reader is also referred to other relevant EANM consensus documents and guidelines [34, 35].
4. Orthopaedics, sports and traumatology [36, 37]
  Periostitis, including shin splints and thigh splints [38]
  Enthesopathies, including plantar fasciitis, Achilles tendinitis and bursitis
  Spondylolisthesis (acute or subacute)
  Radiological occult stress-related fractures (e.g. scaphoid, tarsals) or nonspecific symptoms [39]
  Insufficiency fractures, including osteoporotic vertebral or occult fractures, sacral fractures, femoral head or neck fractures, tibial plateau fractures, tarsal and metatarsal fractures
  Septic loosening, mechanical complication, and synovitis of internal fixation (long bones or spine) or prosthesis (hip, knee, ankle, or shoulder)
  Pseudoarthrosis (delayed union, non-union)
  Periarticular heterotopic ossification
  Viability of bone graft
5. Metabolic bone disease [40–42]
  Hyperparathyroidism (primary and secondary)
  Osteomalacia
  Renal osteodystrophy
  Rare skeletal manifestations of endocrine disorders, including hyperthyroidism and acromegaly
  Vitamin D deficiency
6. Paediatrics
  Osteochondritis of the hip (Legg-Calvé-Perthes disease)
  Transient synovitis of the hip
  Osteoid osteoma
  Battered child syndrome
  Mandibular condylar hyperplasia
  Bone infarction (sickle cell disease, thalassaemia)

### Exploration of unexplained symptoms

1. Subacute or chronic musculoskeletal or bone pain with normal clinical examination and radiographs
  Arthralgia, monoarthritis, oligoarthritis, polyarthritis, localized or multifocal bone pain and backache
2. Further exploration of abnormal biochemical (e.g. phosphate or calcium metabolism) or radiological findings
3. Fever of unknown origin: exclusion of osteomyelitis

### Metabolic assessment prior to initiation of therapy

1. Evaluation of the activity of arthropathies and to confirm active synovitis prior to radiation synovectomy or before infiltration of facet joints with corticosteroids
2. Evaluation of osteoblast activity in case of Paget’s disease before initiating treatment with bisphosphonates
3. Assessment of benign or malignant vertebral compression fracture prior to vertebroplasty or kyphoplasty

### Treatment monitoring

Quantitative bone SPECT/CT is a novel technique with potentially useful applications in treatment response monitoring in bone [43]. However, the exact role in routine clinical practice has yet to be determined.

### Bone scan not indicated

Bone scan may not be the preferred investigation in the following conditions

1. Bone lesions with known inconsistent scintigraphic findings, such as plasmacytoma, multiple myeloma, chordoma, or Ewing’s sarcoma
2. Benign bone lesions and incidentalomas when properly characterized by radiological imaging, including bone island, uncomplicated haemangioma, osteitis condensans ilii, nonossifying fibromas, asymptomatic enchondroma of the long bones, ganglion cyst and asymptomatic Paget’s disease
3. Symptomatic degenerative joint disease well characterized on radiological imaging, properly diagnosed based on the pain syndrome and a well performed clinical examination

Even though bone scintigraphy may in general not be the preferred imaging modality in the conditions listed above, this recommendation should be assessed within the specific clinical context of the patient.

## Specification of the examination procedure

### Qualifications and responsibilities of personnel

All physicians and personnel involved in performing and reporting bone scintigraphy should be sufficiently qualified and experienced in accordance with applicable laws, and individual responsibilities should be documented in standard operating procedures.

### Request

The written or electronic request form should provide sufficient information to demonstrate the medical necessity of the examination. This should include current signs and symptoms, relevant history (skeletal surgery, trauma, or recent radiation or chemotherapy), and the specific reason for the examination or provisional diagnosis. Outpatients should also bring the results of all other relevant examinations that have already been performed (laboratory, radiological, scintigraphic, or other).

### Patient preparation

When making an appointment, the patient should receive information on how the examination is performed and its estimated duration. Patients should be informed that they may eat and drink and of the need to report a pregnancy, any delay in menstrual cycle, or active breastfeeding. Information leaflets and/or displays should be available in the waiting area of the nuclear medicine service, and all information should preferably be accessible through the website of the institution.

Prior to tracer injection, the nuclear medicine physician or technologist must explain the purpose of the examination, the expected benefits, and answer any questions. The patient is informed as to how the examination is to be performed (e.g. multiple planar acquisitions, additional SPECT/CT, etc.), taking into account the specific clinical problem. Relevant information that may assist in interpretation of imaging findings are checked with the patient, including:

1. History of fractures, trauma, osteomyelitis, cellulitis, oedema, arthritis, neoplasms, metabolic bone disease, or limitation of function
2. Current symptoms and physical findings
3. Results of prior bone scintigraphy or other imaging studies such as conventional radiography, CT and MRI (it is strongly recommended that hard copies or computer files of previous examinations be obtained)
4. History of recent nuclear medicine studies
5. Laboratory results
6. History of therapy that might affect the results of bone scintigraphy (see Precautions)
7. History and dates of prior orthopaedic surgery (e.g. presence and location of prosthetic implants)
8. History of anatomical or functional renal/urinary tract abnormalities
9. Contraindications for hydration

At this time, the physical condition of the patient should be assessed and whether current symptoms (e.g. pain, immobility, etc.) may hamper acquisition of optimal images. In patients with severe pain, an appropriate analgesic strategy should be implemented in consultation with the treating/referring physician. In addition, the scanning parameters may be adapted to accommodate the patient (see Protocol/image acquisition).

Unless contraindicated, patients should be well hydrated and instructed to drink one or more litres of water during the time between injection and imaging. All patients should be asked to void their bladder frequently during the time between injection and delayed imaging as well as immediately prior to the scan. The patients should drink a large amount of fluids during the 24 h after radiopharmaceutical administration. In patients on renal replacement therapy, haemodialysis performed from 15 min to 5 h after injection of the radiopharmaceutical can successfully decrease blood pool and soft tissue activity to nearly normal levels [44, 45]. However, careful planning of the examination and consultation with the treating nephrologist are recommended.

### Precautions

#### Pregnancy and lactation

In women of childbearing age, it is necessary to verify the absence of pregnancy. In a patient who is known or suspected to be pregnant, a clinical decision is necessary to consider the benefits against the possible harm of carrying out any procedure. If medically justified, radiation exposure should be delayed until after both pregnancy and breastfeeding. Evaluation by other techniques such as ultrasonography or MRI is preferred. While interruption in breastfeeding is not essential according to the ICRP, this is on the basis that there is no free pertechnetate in the radiopharmaceutical [46]. Therefore, an interruption of at least 4 h during which one meal is discarded is advised to be on the safe side.

#### Possible drug interactions

The main drugs that may interfere with the quality of scintigraphic images are:

1. Aluminium: reduced skeletal tracer uptake, diffuse hepatic tracer uptake, increased renal tracer uptake
2. Androgen deprivation therapy for prostate cancer (bicalutamide, oestrogens): increased mammary tracer uptake in case of gynecomastia
3. Bone-modifying agents (including bisphosphonates and denosumab) or agents interfering with osteoblast function (e.g. cabozantinib): reduced skeletal tracer uptake [47]
4. Corticosteroids: reduced skeletal tracer uptake, reduced tracer uptake at fracture sites
5. Haematopoietic growth factors: increased spinal tracer uptake, possible increased tracer uptake in the appendicular skeleton
6. Iron: increased renal tracer uptake, increased tracer uptake at site of intramuscular injection, diffuse hepatic tracer uptake
7. Methotrexate: diffuse hepatic tracer uptake
8. Nephrotoxic chemotherapy: increased renal tracer uptake and reduced skeletal tracer uptake
9. Nifedipine: reduced skeletal tracer uptake

### Radiopharmaceuticals

#### Physical characteristics of the radionuclide

^99m^Tc decays by isomeric transition with a half-life of 6.02 h to ^99^Tc by emitting one 140.5-keV gamma ray per decay.

#### Characteristics of the tracer molecule

The molecules most commonly used for performing bone scintigraphy are bisphosphonates: methylene diphosphonate (MDP), hydroxymethylene diphosphonate (HMDP) or hydroxyethylene diphosphonate (HDP), and 2,3-dicarboxypropane-1,1-diphosphonate (DPD).

#### Pharmacokinetics

The injected radiolabelled bisphosphonates adsorb to the surface of hydroxyapatite crystals in proportion to local bone vascularization and osteoblastic activity. After intravenous administration, the plasma clearance of bisphosphonates is biexponential and a function of skeletal uptake and urinary elimination. Four hours after injection, approximately 50 to 60 % of the injected amount is fixed in the skeleton, the unbound fraction (34 %) is excreted in the urine, and only 6 % remains in the circulation. Tracer elimination through the gastrointestinal tract is insignificant. Maximum bone accumulation is reached 1 h after tracer injection and remains practically constant up to 72 h.

#### Radiopharmaceutical preparation and storage

The bisphosphonates listed above are commercially available and supplied in a vial containing the bisphosphonate, a stannous reducing agent and other excipients in the form of a powder ready for labelling. Vials containing the sterile nonpyrogenic lyophilisate should be stored at 4 – 8 °C or room temperature, as required by the manufacturer. These kits can be used until the expiry date of the batch (1 to 2 years after the date of manufacture).

The radiopharmaceutical is prepared by the addition of the required amount of sodium pertechnetate [^99m^TcO_4_^−^] diluted in sterile physiological saline to the vial in accordance with the manufacturer’s instructions. After labelling, the preparation should be kept at between 4 °C and 8 °C or at room temperature, depending on the brand used, and remains stable for 8 h. Because the radiopharmaceutical is susceptible to oxidation, care should be taken to avoid introducing air into the multidose vial during preparation or removal of doses. The radiopharmaceutical should be used within 6 h of preparation unless allowed otherwise by the manufacturer.

#### Administered activity

The radiopharmaceutical has to be administered by the intravenous route, e.g. by direct intravenous injection, via an indwelling catheter or using a butterfly needle.

The activity of the radiopharmaceutical to be administered should be determined after taking into account Directive 2013/59/EURATOM of the Council of the European Union, which guarantees health protection of individuals with respect to the dangers of ionizing radiation in the context of medical exposure. According to this directive, member states are required to bring into force such regulations as may be necessary to comply with the directive. One of the criteria is the designation of Diagnostic Reference Levels (DRL) for radiopharmaceuticals; these are defined as levels of activity for groups of standard-sized patients and for broadly defined types of equipment. These levels are expected not to be exceeded for standard procedures when good and normal practice regarding diagnostic and technical performance is applied. For the reasons discussed above, the activities recommended below for ^99m^Tc-bisphosphonate should be considered only a general indication, based on data in the literature and current experience. However, nuclear medicine physicians in each country should respect the DRLs and the rules set out in local laws.

For bone scintigraphy in adults, the average activity administered by a single intravenous injection should be 500 MBq (300 – 740 MBq, 8 – 20 mCi; Table 1). The administered activity usually ranges between 8 and 10 MBq/kg for adults. Lower activities may be used when equipment with higher detector sensitivity or resolution recovery resulting in similar image quality is available. For markedly obese adult patients, the administered activity may be increased to 11 – 13 MBq/kg. If the injected activity falls outside these recommended limits for clinical reasons, the deviation should be kept as small as possible. Practitioners could be required to justify administration of activities greater than local national DRLs.

**Table 1:** Applicable dose reference levels for bone scintigraphy

	Children						Adults
	3.5 kg	10 kg	20 kg	30 kg	40 kg	50 kg	
Activity (MBq)	40	95	170	240	310	375	300 – 740
Effective dose (mSv)	2.0	2.4	2.5	2.6	2.7	2.8	2.9 – 4.0

The activities administered to children should be a fraction of those administered to adults calculated in relation to body weight according to the factors given by the EANM/SNMMI Paediatric Dosage Harmonization Working Group [9, 48]. In children, the EANM recommends a baseline activity of 35 MBq for ^99m^Tc-bisphosphonates (with a minimum activity of 40 MBq) that should be adjusted based on the class of the radiopharmaceutical (class B) and the weight of the child (Table 1).

### Protocol/image acquisition

#### Instrumentation

Single-headed or dual-headed gamma camera equipped with a low-energy, high-resolution parallel-hole collimator is recommended. A low-energy general purpose collimator may alternatively be used for early dynamic and blood pool images. The energy window is centred on the photon energy peak of ^99m^Tc (140 keV) and the window width is generally set at 15 % or 20 %. An asymmetrical window may be used to improve resolution (e.g. 20 % with a 3 % offset) but its use must be supported by a physicist and an appropriately tailored quality assurance programme [49, 50].

#### Planar and whole-body acquisitions

For the vascular phase of the examination, the camera is positioned centred on the area of interest. The dynamic acquisition of 30 to 60 images with a duration of 1 – 2 s each and a matrix of 64 × 64 or 128 × 12 8 pixels is started simultaneously with the intravenous tracer injection. The early and late planar images are focused on one or more region(s). The early images are acquired between 1 min and 10 min after intravenous injection of ^99m^Tc-labelled bisphosphonate, with an acquisition time of 3 min to 5 min and a matrix size of 128 × 128 or 256 × 256. Delayed images are usually acquired 2 h to 5 h after injection of the radiolabelled bisphosphonate using a predefined duration (4 – 10 min) or number of counts, with a matrix size of 128 × 128 or 256 × 256. When using a predefined number of counts, at least 700,000 to 1,000,000 counts are required for scanning the thoracoabdominal region, 250,000 to 400,000 counts for the large joints and skull, and 150,000 to 250,000 for the distal joints, depending on the field of view (FOV) of the gamma camera. The larger the FOV, the larger the number of total counts required to give similar count densities over equivalent regions of the skeleton [51]. Moreover, the presence of organs (typically the kidneys) with physiologically high count densities may hamper visualization of contiguous structures (typically the spine).

A pinhole collimator acquiring high-resolution planar views of a small area can be used to complete the examination, particularly in infants and children. A total count of 50,000 to 100,000 is recommended. Zoom magnification or a converging collimator may also be used to improve resolution. The physician interpreting the image should be notified when a collimator that introduces distortions, such as a pinhole, has been used. Additional projections, such as lateral, oblique, tangential and special views may be obtained if necessary.

Whole-body bone scintigraphy can be accomplished with multiple overlapping (spot) images or with continuous imaging (i.e. whole-body scan) obtained in both anterior and posterior projections. In adults, whole-body studies are currently preferred. In children multiple planar acquisitions are commonly performed, rather than whole-body studies. When spot views are used as the primary acquisition, the regions of skeleton covered by each spot view must overlap to avoid missing any area.

Whole-body images are routinely acquired. Exceptions may include the presence of localized symptoms or patient-specific factors (children, or patients with severe pain or claustrophobia). The recommended scanning speed is 25 – 30 cm/min for early-phase images (if applicable) and 10 – 15 cm/min for delayed acquisitions. The scanning speed should be adjusted so that routine anterior and posterior whole-body images each contain more than 1.5 million counts. The image format is 1,024 × 256 or 2,048 × 512. The whole-body images can be processed with a spatial filter to improve pixel-to-pixel variation.

It is often helpful to increase the time between tracer injection and image acquisition in order to optimize the bone-to-background ratio, if the skeleton is poorly visualized (e.g. renal insufficiency), for the imaging of specific anatomical regions (e.g. the pelvis patients with urinary retention, or the distal extremities in patients with peripheral circulatory disorders), and in older patients with impaired bone metabolism (osteoporosis, osteomalacia). Usually, the goal of these late (6 – 24 h) images is to detect stress fractures, osteitis, osteomyelitis or bone metastases.

#### SPECT and SPECT/CT acquisitions

Diagnostic sensitivity and specificity of bone scanning can be significantly increased by using SPECT or, if available, SPECT/CT. Tomographic images may thus be acquired to assist in localizing anomalies seen on the whole-body images and to improve lesion contrast. In oncology, tomographic images should be used not only for better localization of unclear lesions on planar images but also in patients with a high pretest probability of having bone metastases (for example, in patients with increased tumour marker levels, recurrent cancer, or an advanced tumour that predominantly metastasizes to bone etc.) [22, 52].

SPECT imaging should be performed as recommended by the gamma camera manufacturer. In a typical acquisition protocol for a dual-headed gamma camera with the detector heads oriented in a 180° geometry, a total of 60 or 64 frames per detector head, each with duration of 10 to 30 s are acquired over 360° into a 128 × 128 matrix (pixel size 4.6 × 4.6 mm). An equivalent total number of counts should be acquired if continuous acquisition is used.

SPECT/CT images are acquired using a multimodality camera that combines a gamma camera and a multislice spiral or flat panel/cone beam CT scanner (currently with 1, 2, 4, 6 or 16 slices). The CT scan is performed immediately before or after the SPECT acquisition. The acquisition protocols are specific to each type of machine. The CT component can be performed either for attenuation correction and anatomical localization or as an optimized diagnostic CT scan [53]. If the CT scan is obtained for attenuation correction and anatomical localization only, the use of a low milliampere-seconds setting is recommended to decrease the radiation dose to the patient. However, there are significant differences in operating characteristics between types of scanner, hampering recommendations on absolute values of milliampere-seconds. Operators should be aware of the characteristics particular to their scanner and understand the range of settings that are consistent with meeting the required image quality and reference dose values. The use of intravenous iodinated contrast material is generally not required, and MRI is preferred to assess soft tissue disease.

The image matrix size is 512 × 512, with a tube voltage of 80 – 130 kV and intensity–time product of 2.5 to 300 mAs, depending on the anatomical region being scanned and the dose reduction software used. The pitch can range from 1 to 2 and the slice thickness is generally 0.33 – 2.0 mm for scanning the extremities and 0.33 to 5 mm for the spine (Table 2). The final image is obtained after applying a high-resolution filter. The SPECT/CT acquisition may span a single FOV, usually covering a region of 40 cm, or can include multiple contiguous or separate FOVs.

**Table 2:** Suggested views and image acquisition parameters for skeletal scintigraphy of major joints using multimodality SPECT/CT equipment

Region	Static blood pool		Static delayed^a^		SPECT/CT^b^			
	Indication	Acquisition	Indication	Acquisition	Indication	Acquisition		
						SPECT	Attenuation correction/localization CT	Diagnostic CT
Wrist/hand	Routinely recommended Immediately following the injection of the radiotracer, place both hands (palms down) on the camera	Planar images 256 × 256 matrix LEHR or LEGP collimators 2 – 5 min per view	Routinely recommended	Planar images 256 × 256 matrix LEHR collimators 5 min/500 kcts per view	If planar imaging is nondiagnostic, usually late-phase SPECT/CT	LEHR collimators Matrix 128 × 128 128 angles 20 s/angle Step mode Noncircular rotation	2.5 – 40 mA 80 – 130 keV 1 – 5 mm slice thickness	40 – 335 mA 80 – 130 keV 0.33 – 2.0 mm slice thickness
Hips	Routinely recommended Depending on the size of the replacement, two images may be required to ensure the whole area is covered LEHR or LEGP collimators	Anterior/posterior images 256 × 256 matrix 2 – 5 min per view	Routinely recommended Depending on the size of the replacement, two images may be required to ensure the whole area is covered	Anterior/posterior images 256 × 256 matrix LEHR collimators 5 min/500 kcts per view This may depend on how full the bladder is, for example	If planar imaging is nondiagnostic, usually late-phase SPECT/CT Particularly useful for evaluation of hip prosthesis	LEHR collimators Matrix 128 × 128 128 angles 20 s/angle Step mode Noncircular rotation	2.5 – 40 mA 80 – 130 keV 1 – 5 mm slice thickness	40 – 335 mA 80 – 130 keV 0.33 – 2.0 mm slice thickness
Knee	Routinely recommended Also consider early-phase SPECT/CT might be helpful in inflammation and/or infection	Anterior/posterior images 256 × 256 matrix LEHR or LEGP collimators 2 – 5 min per view	Benign and malignant tumours, orthopaedic (arthroplasty, osteoarthritis, etc.), infection, inflammation, postoperative assessment, problem solving in equivocal conventional imaging	Anterior/posterior images Lateral images 256 × 256 matrix LEHR collimators 5 min/500 kcts per view This may depend on how full the bladder is, for example	If planar imaging is nondiagnostic, usually late-phase SPECT/CT Particularly useful for the evaluation of knee prosthesis	LEHR collimators Matrix 128 × 128 128 angles 20 s/angle Step mode Noncircular rotation	2.5 – 40 mA 80 – 130 keV 1 – 5 mm slice thickness	40 – 335 mA 80 – 130 keV 0.33 – 2.0 mm slice thickness
Spine	Radiologically inconclusive result for fracture with red flags Suspicion of infection, including spondylitis and spondylodiscitis In case of likely fractures to discriminate recent from old fracture In the postsurgery setting (with or without implants)	Anterior/posterior images 256 × 256 matrix LEHR or LEGP collimators 2 – 5 min per view	Routinely recommended	Anterior/posterior images 256 × 256 matrix LEHR collimators 5 min/500 kcts per view	If planar imaging is nondiagnostic, usually late-phase SPECT/CT To help identify patients with low back pain who would benefit from facet joint infiltration In the postsurgery setting (with or without implants)	LEHR collimators Matrix 128 × 128 60 – 128 angles 15 – 20 se/angle Step mode Noncircular rotation	2.5 – 40 mA 80 – 130 keV 1 – 5 mm slice thickness	40 – 335 mA 80 – 130 keV 0.33 – 2.0 mm slice thickness

^a^Whole-body image acquisition should be considered in patients with suspected malignancy (metastatic, primary), rheumatoid arthritis, elevated alkaline phosphatase level, metabolic bone disorders, hyperparathyroidism (brown tumours, superscan, soft tissue uptake in lung, stomach, kidneys, heart or pancreas), infectious disease

^b^CT settings are variable depending on local requirements/protocols/cameras etc. The use of dose reduction features is recommended if available

#### Image processing

Planar images do not require particular processing. It should be noted that current digital gamma cameras and workstations allow changing the range of image contrast, improving the diagnostic value of the images. In addition, this allows the optimum contrast to be chosen for printing the images that will be sent to the requesting clinician. A relative image quantification may be performed on certain areas.

In the case of SPECT one should take into account the different types of gamma camera and software available. Reconstructions are preferably performed using the three-dimensional (3D) iterative ordered subsets expectation maximization algorithm (OSEM), including classical corrections for attenuation and scatter, and nowadays also resolution recovery. The parameters used for reconstruction may vary between vendors, but must permit a good image resolution while maintaining adequate noise levels (using postprocessing). Typically, three or five iterations are necessary and eight to ten subsets. Postprocessing is usually performed using a gaussian filter (width at half-maximum of 4 to 10 mm), or a Butterworth filter (conventional parameters 10/0.5). Novel reconstruction algorithms, including multimodal reconstruction, are being introduced and alternatively can be used.

With a multimodality SPECT/CT system, coregistered and aligned CT images, SPECT images and SPECT/CT fused images can be generated and visualized. The 3D images are usually displayed as 2D orthogonal (axial, coronal and sagittal) and maximum intensity projections. SPECT images with and without attenuation correction should be available for review. Care must be taken when judging attenuation-corrected images since even slight misalignment between the SPECT and CT images may cause attenuation and reconstruction artefacts, which can lead to overestimation or underestimation of the tracer uptake in a certain region. Typical parameters used by the main multimodality camera manufacturers are shown in Table 3.

**Table 3:** Parameters from the three main multimodality camera manufacturers

	Manufacturer		
	General electric	Philips	Siemens
Reconstruction algorithm	OSEM 3D	OSEM 3D	OSEM 3D
Attenuation and scatter correction	Yes	Yes	Yes
Resolution recovery	Evolution	Astonish	Flash 3D
Parameters	Three iterations	Three iterations	Five iterations
	Ten subsets	Ten subsets	Eight subsets
	Butterworth filter (10/0.5)	Hanning filter (threshold 1.73)	Gaussian filter (4 mm)

#### Adapting scan parameters

The suggested imaging parameters may be adapted in consideration of the specific clinical condition of the patient (e.g. pain due to bone metastases, vertebral fractures, loosening prosthesis, etc.). In such circumstances, shortening the duration of the examination by decreasing the time between injection and imaging, reducing the acquisition time, or increasing the administered activity can be considered.

#### Selecting the appropriate image acquisition technique

1. Sequential planar acquisitions are used in the assessment of various diseases: infectious or inflammatory diseases, trauma, malignancy or pain syndromes affecting the extremities.
2. Whole-body bone scintigraphy is routinely used in oncology and other settings. In those cases, limited bone scan or spot views are indicated only where an uptake abnormality or an equivocal finding detected on whole-body image needs to be clarified.
3. Pinhole collimator acquisitions are preferentially reserved for studies in children, and in particular for scanning the hips (osteochondritis, aseptic necrosis).
4. Multimodality SPECT/CT imaging is indicated for the assessment of lesions equivocal on planar bone scintigraphy or localized pain syndromes with normal findings on planar scintigraphy, in particular in the staging of malignancies that have a tendency to metastasize to bone [54]. SPECT/CT can also be used in patients with multiple equivocal benign lesions in the axial or appendicular skeleton to increase specificity and diagnostic certainty.

#### Indications for SPECT/CT imaging

The indications for SPECT/CT imaging in daily clinical practice are broad and include (but may not be limited to) the following:

1. Oncology: in case of abnormal planar scintigraphy to improve lesion localization
2. Suspected traumatic injuries of the axial or appendicular skeleton
3. Assessment of lesions in the tarsal or carpal small bones, in particular after trauma
4. Suspicion of axial or peripheral osteoid osteoma
5. Assessment of the spine and sacroiliac joints in case of rheumatic disorders
6. Diagnosis of osteonecrosis and bone infarction
7. Diagnosis of infectious lesions, such as osteomyelitis and spondylodiscitis (complemented with infection imaging)
8. Diagnosis of tendinitis
9. Evaluation of painful prosthesis
10. Evaluation of residual pain after orthopaedic surgery on the axial or peripheral skeleton
11. Assessment of malignant or pseudomalignant lesions
12. Exploration of extraskeletal pathology or uptake

### Interpretation

#### Normal distribution of radiolabelled bisphosphonates

**Fig. 1:**
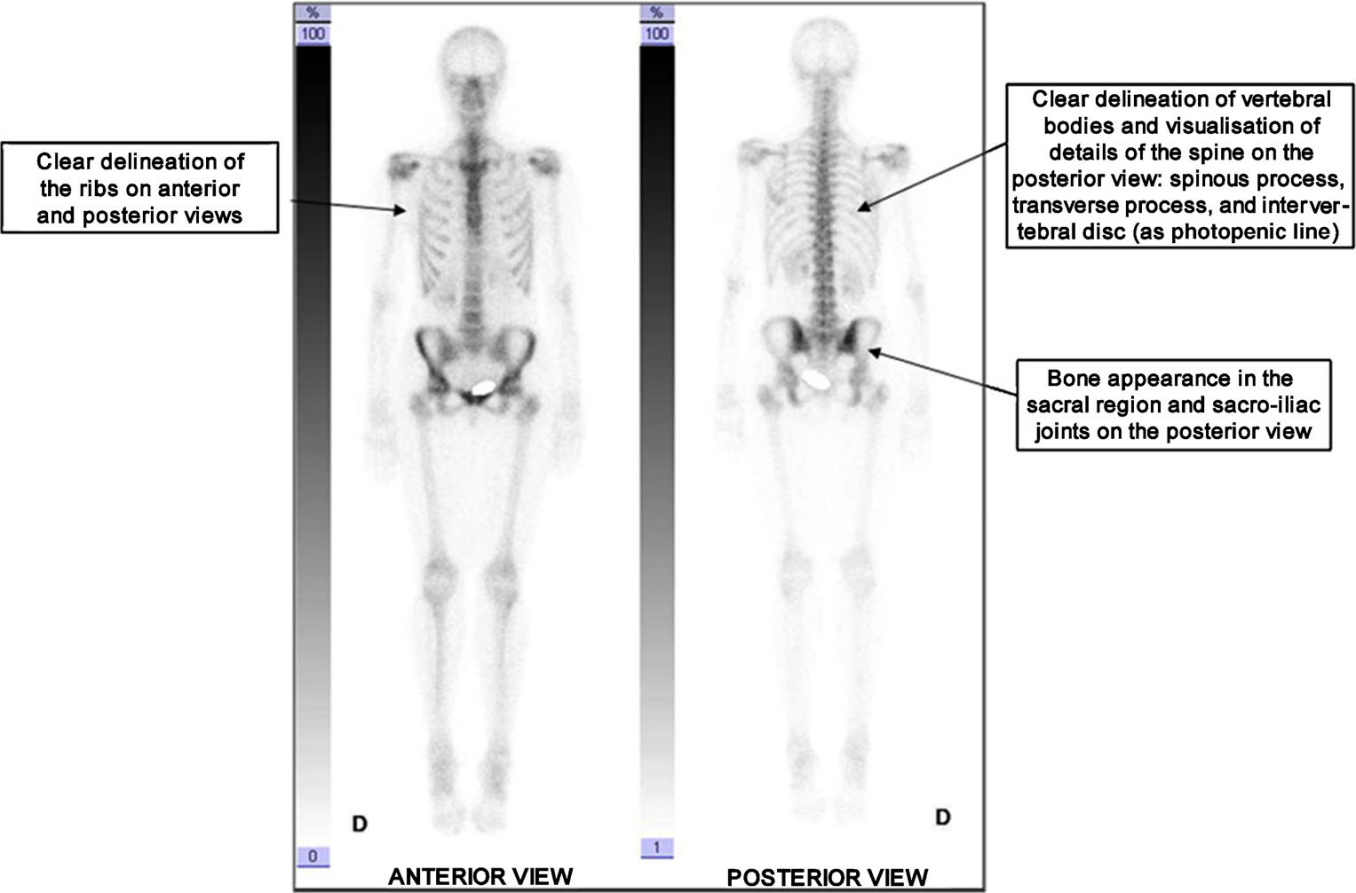
Normal whole-body scan. Scintigraphic criteria allowing assessment of the quality and interpretability of a whole-body scan

Bone scintigraphy is a very sensitive method for localization of skeletal diseases, but the specificity may be low. Skeletal or joint abnormalities should be interpreted taking into account all available information, especially patient history, recent findings, physical examination and other test or examination results. Correct image interpretation requires detailed knowledge of the normal distribution of radiolabelled bisphosphonates. Particular attention should be paid to the symmetry and homogeneity of tracer uptake. Image quality should be assessed before starting to report scan findings (Fig. 1).

#### Bone abnormalities

Both increases and decreases in tracer uptake have to be assessed and any abnormality can be either focal or diffuse. Focal or diffuse increased skeletal uptake can be objectively assessed by comparison with the contralateral bone or soft tissue. The localization, size, shape, intensity, and number of abnormal findings should be described. In comparison to the normal bone activity, increased tracer uptake indicates increased osteoblastic activity. Some osteolytic skeletal lesions appear as a region of reduced tracer uptake, either surrounded by a rim of increased tracer deposition or, conversely, with a punched-out appearance. Decreased uptake is less common than focally increased activity and sometimes hard to identify. Indeed, bone scintigraphy has a low diagnostic sensitivity for purely osteolytic lesions (e.g. multiple myeloma).

Differential diagnosis can sometimes be based on the configuration, location and number of abnormalities, although most patterns are nonspecific. Lesions detected on bone scintigraphy can take an extended time to normalize, reflecting the protracted course of bone healing which may take many months. Therefore, it is rarely useful to repeat an examination within 4 or 6 months. In oncology, a decrease in intensity of tracer uptake and in the number of abnormalities often indicates improvement or may be secondary to focal therapy (e.g. radiation therapy). An increase in the intensity or the number of foci of increased uptake on scans performed less than 6 months apart may represent disease progression, but can also be associated with a flare phenomenon [55].

#### Soft tissues findings

The renal system and urinary tract are also normally visualized on the examination, as well as diffuse or focal tracer uptake in the soft tissues. Tracer uptake in the kidney can be focal or diffuse. A diffusely increased soft tissue uptake can be caused by drug interference, failed ^99m^Tc labelling, severe osteoporosis, renal failure, dehydration, or an insufficiently long interval between tracer injection and image acquisition. Conversely, a low or absent tracer uptake in the soft tissues may be caused by an excessive avidity for the tracer of osteoblasts populating the axial skeleton, resulting in a “super bone scan” appearance or an excessively long interval between tracer injection and imaging.

#### Sources of error

Focal soft tissue hot spots have a wide range of causes, and this may lead to an incorrect diagnosis of skeletal disease on planar imaging. In striated muscle, for example, the causes of increased uptake include the following:

- Repeated intramuscular injections of iron supplements
- Haematoma/necrosis/sickle cell anaemia
- Rhabdomyolysis (mechanical, toxic, electrical, …)
- Muscular abscess
- Primary tumours (rhabdomyosarcoma, other sarcomas)
- Metastases from solid tumours
- Poly(dermato)myositis (many causes)
- Severe renal insufficiency/hypercalcaemia/malignant calcinosis/multiple myeloma
- Myositis ossificans

Attenuation artefacts caused by metal parts or motion artefacts are generally obvious, as is tracer extravasation at the injection site due to (partly) paravenous injection.The most common artefacts are related to urinary tracer contamination in patients with dilatation, stasis or an anatomical variant of the urinary tract, especially after urological surgery, or contamination during urination. Imaging artefacts include the following:

- Injection artefacts.
- Imaging too early after injection, before the radiopharmaceutical has been optimally cleared from soft tissues.
- Greater than necessary collimator to patient distance.
- Patient movement.
- Pubic or sacral lesions obscured by superposition of bladder activity.
- Urine contamination or a urinary diversion reservoir.
- Changing bladder activity during SPECT of the pelvic region.
- Purely lytic lesions with decreased or absent tracer uptake.
- Prosthetic implants, radiographic contrast materials or other attenuating artefacts which may obscure normal structures.
- Homogeneously increased bony activity (e.g. ‘superscan’).
- Renal failure.
- Restraint artefacts caused by soft tissue compression.
- Significant findings outside the area of interest may be missed if a limited study is performed.
- Prior administration of a higher-energy radionuclide, or of a preceding examination with another ^99m^Tc radiopharmaceutical that accumulates in an organ that could obscure or confound skeletal activity.
- Radiopharmaceutical degradation.

Some bone lesions may be purely or predominantly lytic and barely visible on planar bone scintigraphy when they are small (<2 cm): multiple myeloma, infarction, osteonecrosis, haemangioma, or lytic bone metastases. These artefacts and sources of error may be avoided by an additional SPECT/CT acquisition.

## Documentation/reporting

### Clinical context

The nuclear medicine physician should record a brief summary of the reason for the examination, the clinical problem, the medical or surgical history, all relevant laboratory results and radiological findings, and the treatments targeting or interfering with the osteoarticular system.

### Procedure

Technical information should include the international nonproprietary name (INN) of the radiolabelled bisphosphonate, the injected activity in megabecquerels, the site and time of injection, the time of image acquisition, the scanning protocol used (early phase, late phase, SPECT/CT images), the different image acquisitions, and the dose–length product (DLP) and the CT dose index (CTDI) if SPECT/CT is performed. Optionally, the model and installation date of the camera can be stated. Also, any specific patient preparation should be reported (analgesics, anxiolytics, catheter, etc.), in addition to any incidents during imaging and any technical limitations of the examination. The description of the different images acquired during the examination includes details on early-phase images, specific late-phase images, and whole-body acquisitions. When multimodality SPECT/CT imaging is performed, the description of osteoarticular structures focuses on the SPECT images and the SPECT/CT fusion images.

### Findings

Abnormal tracer uptake (increased, decreased, abnormal pattern, bone findings, soft tissue findings) should be clearly stated. If the study is a repeated examination, data are described in correlation with other diagnostic results and in comparison with previous studies. Any skeletal anomalies that are only seen on the CT part of the examination are also reported. Likewise, other relevant nonskeletal pathology detected on the CT images should be mentioned. Software-based assessment of bone abnormalities can assist in reporting, but should not replace assessment by a nuclear medicine physician.

### Interpretation

The conclusion of the report should answer the question posed by the referring clinician and should mention any associated diagnoses. A clear diagnosis should be given if possible, accompanied by a description of the study limitations when appropriate. If the findings on scintigraphy or multimodality imaging are nonspecific, a differential diagnosis should be made, whenever possible mentioning the likelihood of the listed diagnoses. When there is doubt as to the diagnosis or further work-up is required, the nuclear medicine physician may recommend additional tests (laboratory, imaging, biopsy, etc.), especially if several differential diagnoses, which could lead to different clinical management decisions, are possible.

If the scintigraphic findings and/or CT results reveal a life-threatening disease or a condition that requires immediate action, it is the nuclear medicine physician’s responsibility to contact the referring clinician and organize urgent further care.

## Quality control and improvement

### Gamma camera quality control

A strict quality control programme should be routinely performed [50] according to the rules of each country, as stated in Council Directive 2013/59/EURATOM.

### Quality control of the radiopharmaceutical

The radioactive concentration should be determined by measuring the activity of the vial in a calibrated ionization chamber. Labelling efficiency should be >95 %. The manufacturer’s instructions for assessment of radiochemical purity (e.g. by thin-layer chromatography) and local laws should be followed.

## General safety procedures

### Infection control, and patient education concerns

The injection must comply with local applicable guidelines and recommendations. The needle used for tracer injection must be collected in a needle container. Vials, syringes, injection needles and gloves used for injection are stored in lead-shielded containers until safe radioactive levels are attained (see Radiation protection). Side effects or incidents should be reported in accordance with applicable laws.

### Radiation safety

#### Physiological distribution of ^99m^Tc-phosphonates

Phosphonates concentrate in the mineral part of bone, nearly two thirds in hydroxyapatite crystals and one third in calcium phosphate. Two major factors control the accumulation of phosphonates in bone, namely blood flow and extraction efficiency, which in turn depend on capillary permeability, acid–base balance, parathyroid hormone levels, etc. Peak activity through the kidneys is reached after approximately 20 min.

#### Radiation dosimetry

The organ that receives the largest dose of radiation is bone (see table of adsorbed doses, ICRP no. 80, 1998) [56]. The estimated adsorbed radiation doses to various organs in healthy subjects following administration of ^99m^Tc-labelled phosphates and phosphonates are given in Table 4. It is assumed that 50 % of the injected activity is absorbed by the skeleton with an uptake half-life of 15 min, 15 % of the injected activity is retained in the skeleton with a clearance half-life of 2 h, and the remaining 35 % of the injected activity shows a clearance half-life of 3 days. In children, bone uptake concentrates in the metaphyseal growth zones, and this can give rise to absorbed doses in these areas which are larger than the average skeletal dose. In children with disease involving higher uptake in bone and with severely impaired or without renal clearance, the absorbed doses to bone surfaces and bone marrow can increase by a factor of 2, resulting in an increase in the effective dose by approximately 12 %.

**Table 4:** Absorbed doses to various organs in healthy subjects following administration of ^99m^Tc-labelled phosphates and phosphonates according to ICRP Publication 80

Organ	Absorbed dose per unit activity administered (mGy/MBq)		
	Adult	15 years	5 years
Adrenals	0.0021	0.0027	0.0058
Bladder	0.048	0.060	0.073
Bone surfaces	0.063	0.082	0.22
Brain	0.0017	0.0021	0.0043
Breast	0.00071	0.00089	0.0022
Gallbladder	0.0014	0.0019	0.0042
Stomach	0.0012	0.0015	0.0035
Small intestine	0.0023	0.0029	0.0053
Colon	0.0027	0.0034	0.0061
Heart	0.0012	0.0016	0.0034
Kidneys	0.0073	0.0088	0.018
Liver	0.0012	0.0016	0.0036
Lungs	0.0013	0.0016	0.0036
Muscles	0.0019	0.0023	0.0044
Oesophagus	0.0010	0.0013	0.0030
Ovaries	0.0036	0.0046	0.0070
Pancreas	0.0016	0.0020	0.0045
Red marrow	0.0092	0.010	0.033
Skin	0.0010	0.0013	0.0029
Spleen	0.0014	0.0018	0.0045
Testes	0.0024	0.0033	0.0058
Thymus	0.0010	0.0013	0.0030
Thyroid	0.0013	0.0016	0.0035
Uterus	0.0063	0.0076	0.011
Remaining organs	0.0019	0.0023	0.0045
Effective dose (mSv/MBq)	0.0057	0.0070	0.014

The effective dose for an adult is in the order of 3 – 4 mSv and in children 2.5 mSv (Table 1). The additional dose from the CT scan is not taken into account in these figures. The effective dose from the CT scan depends strongly on the machine used: for a diagnostic scan of the hips and spine the effective dose can range between 4 and 10 mSv [57, 58]. The much lower milliampere-second values used for localization or attenuation correction CT scans result in maximum effective doses of 3 mSv. The effective doses for both types of CT scan to the extremities are all less than 0.1 mSv.

#### Radiation protection

Staff radioprotection measures should follow the recommendations for good practice (lead castle, syringe shields, wearing gloves during tracer preparation and injection, etc.). The exposure of caregivers on hospital wards is very low, and no data are available to recommend any specific safety measures, apart from those aimed at limiting contamination. It is recommended that disposable gloves are worn for personal care during the 24 h following the administration of radiopharmaceuticals labelled with ^99m^Tc. Urine and feces can be disposed of into the toilet. Pads, catheters and containers should be handled with gloves. If hospital waste management accepts only materials free of radioactivity, it is recommended that for hospitalized patients all solid waste is collected for 3 days and kept in storage for 4 days to allow sufficient decay of radioactivity. For the patient’s family, no special measures are required.

If tracer is administered erroneously to a patient for whom the radiopharmaceutical was not intended, it is recommended that the patient is sufficiently hydrated and encouraged to urinate frequently, in order to limit the radiation dose to the bladder and pelvis. No special radioprotection measures are required if a patient dies.

## Issues requiring further clarification

The role of ^99m^Tc-phosphonate bone scintigraphy in follow-up of treated cancer patients is still a matter of discussion. There is general agreement that bone scintigraphy is indicated in symptomatic patients. However, it is unproven whether bone scintigraphy is cost-effective in all asymptomatic patients at risk of metastases (with worse prognostic factors). It has not yet been established which subgroups of patients at high risk of metastases may benefit from periodic bone scan examinations. The list of indications for SPECT/CT has not yet fully matured and more clinical trials are required before further evidence-based guidelines can be produced. In addition, the optimal tube current and voltage settings for CT scans performed for localization and attenuation correction remain poorly defined and nonstandardized, requiring further study.

## Disclaimer

The EANM wrote and approved these guidelines to promote the use of high-quality nuclear medicine procedures. These general recommendations cannot be applied to all patients in all practice settings. The guidelines should not be deemed inclusive of all proper procedures and exclusive of other procedures reasonably directed towards obtaining the same results. The spectrum of patients seen in a specialized practice setting may be different from the spectrum usually seen in a more general setting. The appropriateness of a procedure will depend in part on the prevalence of disease in the patient population. In addition, resources available for patient care may vary greatly from one European country or one medical facility to another. For these reasons, the guidelines cannot be rigidly applied.
